# Compressing the lumbar nerve root changes the frequency-associated cerebral amplitude of fluctuations in patients with low back/leg pain

**DOI:** 10.1038/s41598-019-38721-5

**Published:** 2019-02-19

**Authors:** Fuqing Zhou, Yanlin Zhao, Li Zhu, Jian Jiang, Muhua Huang, Yong Zhang, Ying Zhuang, Honghan Gong

**Affiliations:** 1 Department of Radiology, The First Affiliated Hospital, Nanchang University, Nanchang, 330006 People’s Republic of China; 2 School of Information Engineering, Nanchang University, Nanchang, 330031 People’s Republic of China; 3 Department of Pain Clinic, The First Affiliated Hospital, Nanchang University, Nanchang, Jiangxi Province 330006 People’s Republic of China; 4 Department of Oncology, The Second Hospital of Nanchang, Nanchang, 330003 People’s Republic of China; 5 Neuroimaging Lab, Jiangxi Province Medical Imaging Research Institute, Nanchang, 330006 People’s Republic of China

## Abstract

Understanding the central mechanisms responsible for lumbar nerve root compression may facilitate the development of new therapeutic strategies. In this study, our aim was to investigate the amplitude of fluctuations (AF) in five specific frequency bands and the full-frequency band realm to provide novel insight into the rhythm of the neuronal activity of low back/leg pain (LBLP) patients (n = 25). Compared with healthy controls, LBLP patients exhibited a significantly altered AF in multiple brain regions, including the right or left middle and inferior temporal gyri, bilateral precuneus, right anterior insula/frontal operculum, right or left inferior parietal lobule/postcentral gyrus, and other locations at five specific frequencies *(P* < *0*.*01*, *with Gaussian random field theory correction)*. Trends of an increase and a decrease in the AF in pain- and sensory-related regions, respectively, were also observed from low to high frequencies (*Bonferroni-corrected α level of P* < *0*.*05/84*). In addition, in the bilateral rectal gyrus, a significant association was identified between the AF in the five specific frequency bands and disease status (*P* < 0.05). These findings suggest that in LBLP patients, intrinsic functional plasticity related to low back pain, leg pain and numbness affects the AF of the pain matrix and sensory-processing regions in both low- and high-frequency bands.

## Introduction

Low back pain is an extremely common disorder characterized by acute episodes of pain or chronic pain that is associated with disability in middle-aged and elderly persons. Low back pain affects millions of individuals each year^1^. Although the pain is often caused by a peripheral event, there is increasing concern regarding associated brain changes such as structural impairment in specific cortical and subcortical areas (e.g., white matter structural damage^2–4^ or cortical atrophy^5,6^ and thinning^7^). Increased activation in several brain regions of the pain matrix, such as primary sensory cortex (S1) and secondary sensory cortex (S2)^8^, the insula and the prefrontal cortices^9,10^, and disrupted connectivity in the default mode network (DMN) have also been shown^11–14^. In addition, central sensitization has been observed in a subgroup of patients with low back pain and associated brain changes.

Lumbar disc herniation (LDH) is a common cause of low back pain. Approximately 60% of patients with low back pain due to LDH also experience radiating pain to the leg(s). Low back/leg pain (LBLP) due to LDH (LBLP hereafter) in patients is associated with poorer recovery than is observed in patients with low back pain alone^15^. In fact, approximately one-quarter to one-third of these LBLP patients continue to exhibit pain after surgery or failed back surgery syndrome^16^. Leg pain intensity and paresthesia coverage are the primary and secondary outcomes/prognostic factors, respectively^15–17^. Therefore, understanding the role of neural plasticity or the rewiring of the brain in patients with LBLP will improve our knowledge of pain symptoms and the mechanisms underlying LBLP-associated central modulation. In the resting brain, LBLP patients process spontaneous background pain and paresthesia (numbness) stimuli continuously; these stimuli interact with and modulate other intrinsic brain networks through numerous oscillatory waves that, together, constitute a complex dynamic system^12,18^, which can be studied using blood-oxygenation level-dependent (BOLD) functional magnetic resonance imaging (fMRI). According Buzsáki’s framework^7^, oscillatory BOLD waves can be divided into the following five specific frequency bands: slow-6 (0–0.01 Hz), slow-5 (0.01–0.027 Hz), slow-4 (0.027–0.073 Hz), slow-3 (0.073–0.198 Hz) and slow-2 (0.198–0.25 Hz). Oscillations within specific low-frequency ranges (typically defined as 0.01–0.1 Hz) have been shown to be linked with a variety of neural processes, including input selection, plasticity, binding and consolidation and the cyclic modulation of gross cortical excitability and long-distance neuronal synchronization^12,18–20^. Although controversial, alterations in relatively high-frequency bands have been shown in the resting state and, thus, may be of physiological importance in pain^21,22^, including chronic somatic pain^23^, visceral pain^24^ and fibromyalgia^25^.

In accordance with the above findings, we hypothesize that the dual-stimulation (pain and numbness) in LBLP may contribute to the alterations of neuronal activity in the low- and high-frequency bands of BOLD waves. In the present study, we examined the amplitude of fluctuations (AF) of intrinsic oscillations in five specific frequency bands and full-frequency bands (divided into 84 narrow band bins, 0.003 Hz/bin) in LBLP patients compared with healthy controls. In view of the currently available data on idiopathic chronic low back pain^11–14^, we have reason to believe that the relatively high-frequency bands of BOLD waves can be modulated by dual-stimulation (pain and numbness) and, thus, may be of physiological importance in LBLP. We investigated the brain regions with altered AF in the five specific frequency bands and studied their correlations with disease severity and duration, pain level based on a visual analogue scale (VAS) and tactile discrimination ability (a cortical sensory task) in LBLP patients.

## Results

### Clinical characteristics and indices

Two LBLP patients were excluded due to a vascular malformation and an infarction, and 4 LBLP patients and 3 healthy subjects were excluded due to head motion. Ultimately, a total of 25 LBLP patients and 27 healthy control (HC) subjects were selected for group comparisons. All the 25 LBLP patients presented with low back pain (*duration: 37*.*08* ± *10*.*23 months*); 23 (92%) of these patients also presented with left or right leg pain and numbness; and 2 (8%) of these patients also presented with bilateral leg pain. There were no significant differences in age (*55*.*16* ± *1*.*83 vs*. *52*.*96* ± *1*.*63 years; P* = *0*.*7*2*7*), sex (M/F: *13/12 vs*. *15/12*, *χ*^*2*^ *test*, *P* = *0*.*84*) or mean head motion by framewise displacement (Jenkinson) (*0*.*098 *±* 0*.*053 vs*. *0*.*109* *±* *0*.*049; P* = *0*.*449*) between the LBLP patients and the HCs. Compared with HCs, the LBLP patients showed significantly lower scores in the Japanese Orthopaedic Association (JOA) Back Pain Evaluation (*13*.*72* ± *1*.*13 vs*. *28*.*96* ± *0*.*04*, *P* < *0*.*0001*) and higher VAS scores (*5*.*78* ± *0*.*21 vs*. *0* ± *0*, *P* < *0*.*0001*). In the LBLP patients, decreased performance on the two-point tactile discrimination (TPTD) test was observed in the right (30.8 ± 1.65 mm) and left (31.1 ± 1.21 mm) feet and right (25.3 ± 1.23 mm) and left (26.1 ± 1.25 mm) hands.

### Disease-related differences in the AF within specific frequency bands and full-frequency bands

**Figure 1:**
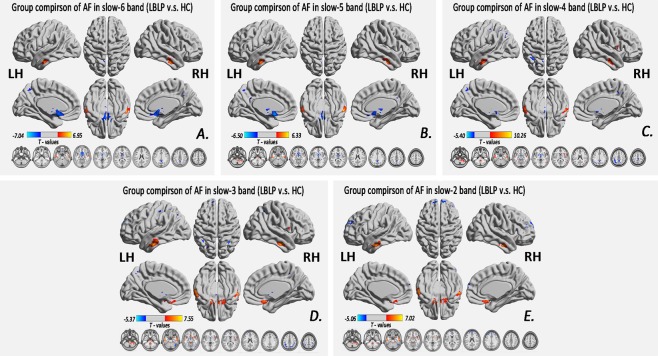
Group comparisons of the AF in specific frequency bands between the LBLP patients and HCs (two-tailed, voxel-level P < 0.01, GRF correction, cluster-level P < 0.05). Note: A-E show alterations in the spatial patterns of the AF in five specific frequency bands: slow-6 (0–0.01 Hz), slow-5 (0.01–0.027 Hz), slow-4 (0.027–0.073 Hz), slow-3 (0.073–0.198 Hz) and slow-2 (0.198–0.25 Hz).

**Figure 2:**
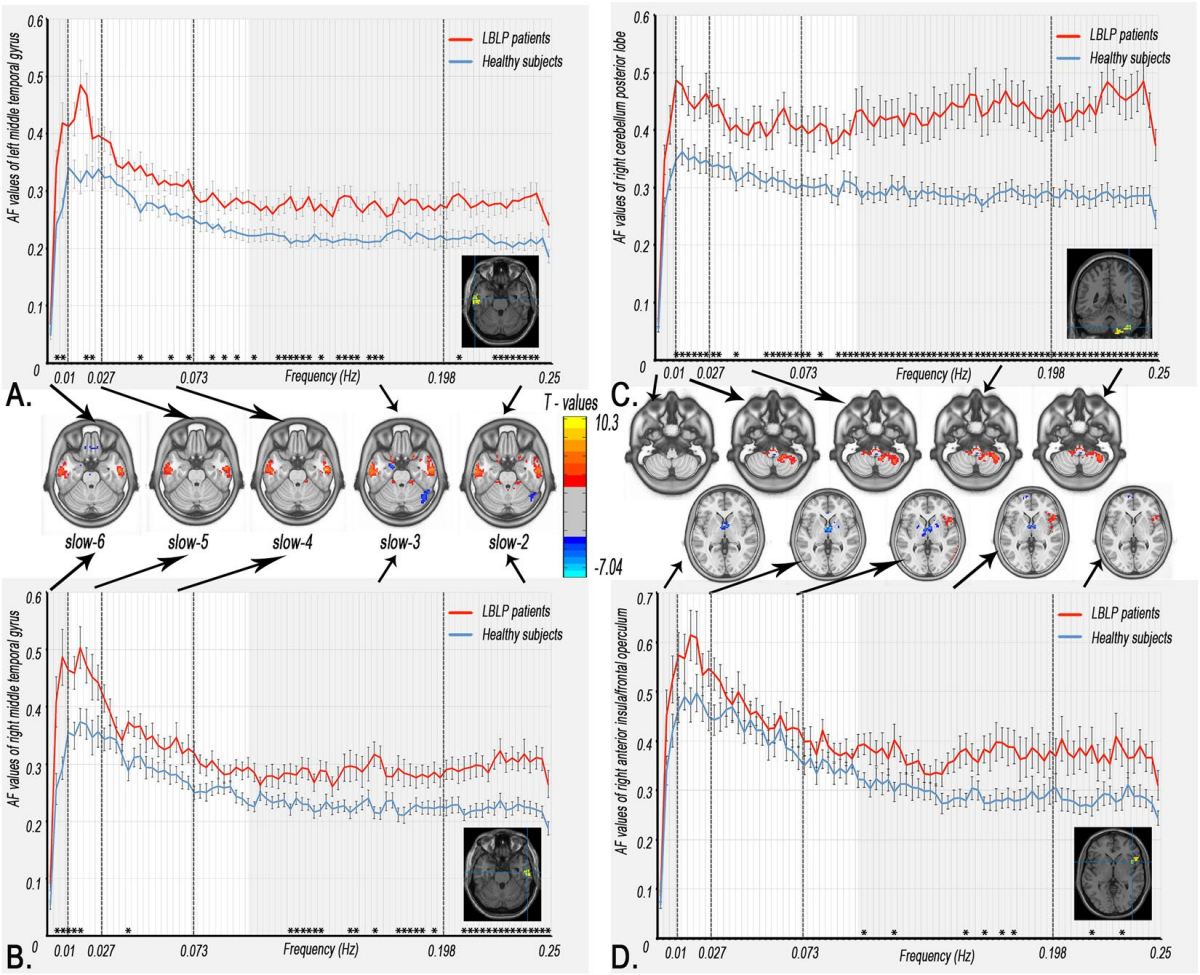
Dynamic changes in the AF in the regions with prominent alterations in five specific frequency bands. The curves indicate the trend in the AF across the full-frequency band (0–0.25 Hz). Note: The red and indigo-blue lines represent LBLP and HC, respectively. The stars at the bottom of the graph represent statistical significance (Bonferroni-corrected α level of P < 0.05/84). The full*-*frequency band (0–0.25 Hz) was divided into 84 narrow band bins (0.003 Hz/bins), and the bright regions represent the conventional frequency band (0.01–0.10 Hz). The brain maps in the middle row show group differences in amplitude between the two groups in the five specific frequency bands (same as Fig. 3).

**Figure 3:**
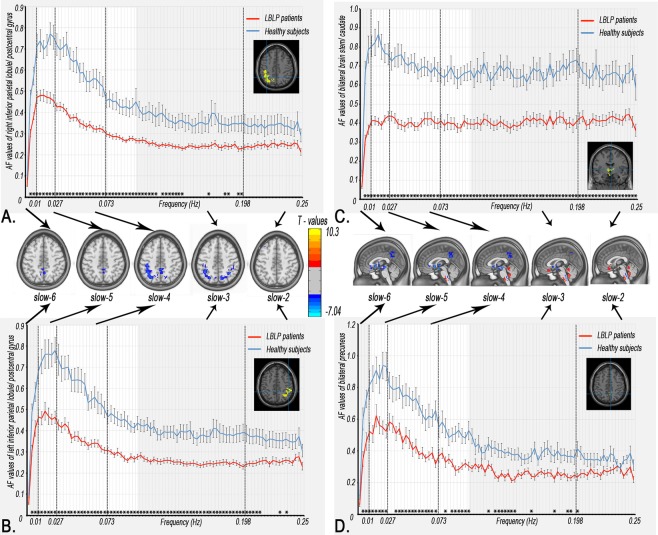
Dynamic changes in AF in the regions showing decreasing results in the five specific frequency bands. These curves indicate the AF change trend across the full-frequency band (0–0.25 Hz).

Figure. 1 and Table 1 show the alteration of the spatial patterns of the AF in the five frequency bands based on voxel-based analyses in the LBLP patients and HCs. The regions with alterations in the five frequency bands were selected as regions of interest (ROIs) for full-frequency band analyses, including the right and left middle and inferior temporal gyri (MTG/ITG), bilateral precuneus (PCUN), right anterior insula/frontal operculum (aINS/fO), right or left inferior parietal lobule/postcentral gyrus (IPL/PoCG), posterior lobe of the right cerebellum (CPL) and bilateral caudate (CAU) *(two-tailed*, *voxel-level P* < *0*.*01*, *Gaussian random field (GRF) theory correction*, *cluster-level P* < *0*.*05)*. Compared with HCs, the LBLP patients consistently exhibited a trend showing an increase in the AF in the right CPL (Fig. 2) and a decrease in the AF in the bilateral PCUN, CAU and right IPL/PoCG (Fig. 3) from the low- to high-frequency bands (*two-tailed t-test*, *Bonferroni-corrected α level of P* < *0*.*00059*, *(P* < *0*.*05/84)*).

**Table 1:** Significant disease-related differences in the AF of specific frequency bands between the LBLP patients and HCs (two-tailed, voxel-level *P* < 0.01, GRF correction, cluster-level *P* < 0.05).

Brain regions	BA	Peak T-scores	MNI coordinates			Cluster size (voxels)
			x	y	z	
**Altered AF in the slow-6 (0–0.01 Hz) band (LBLP** ***vs***. **HC)**						
Right middle and inferior temporal gyri (MTG/ITG)	20,21	6.949	54	−09	−27	151
Left MTG/ITG	20,21	5.026	−60	−15	−27	107
Bilateral brainstem/caudate/thalami/anterior cingulate cortex	25,47	−7.042	0	−3	−18	530
Bilateral precuneus	7,31	−4.187	3	−48	36	133
**Altered AF in the slow-5 (0.01–0.027 Hz) band (LBLP** ***vs***. **HC)**						
Right cerebellum posterior lobe and brainstem		5.218	18	−48	−51	288
Left MTG/ITG	20,21	5.080	−63	−12	−24	165
Right MTG/ITG	20,21	6.333	57	−12	−24	125
Bilateral caudate/anterior cingulate cortex		−6.499	0	−13	−18	397
Bilateral precuneus	7,31	−4.315	0	−51	33	122
**Altered AF in the slow-4 (0.027–0.073 Hz) band (LBLP** ***vs***. **HC)**						
Right cerebellum posterior lobe and brainstem		6.333	3	−36	−57	376
Right MTG/ITG	20,21	10.256	54	−12	−27	150
Left MTG/ITG	20,21	5.992	−51	−15	−27	189
Bilateral brainstem/caudate/thalami		−5.405	0	−3	−18	266
Right temporal–occipital junction (TOJ)	19,37,21	5.028	69	−45	−6	109
Right anterior insula/frontal operculum (aINS/fO)	13,47	5.238	54	21	0	175
Bilateral precuneus	7,31	−4.465	0	−45	45	142
Left inferior parietal lobule/postcentral gyrus (IPL/PoCG)	40,7	−4.777	−48	−51	36	375
**Altered AF in the slow-3 (0.073–0.167 Hz) band (LBLP** ***vs***. **HC)**						
Right cerebellum posterior lobe and brainstem		5.985	21	−45	−57	796
Left MTG/ITG	20,21	6.263	−60	−6	−30	182
Right MTG/ITG	20,21	7.546	54	−12	−27	159
Bilateral rectal gyrus	11,25,47	6.699	6	15	−24	289
Bilateral caudate/thalami		−5.366	−6	−3	0	98
Right aINS/fO	13,47	5.750	36	12	−15	219
Left superior frontal gyrus	10	−5.085	−12	60	27	97
Bilateral precuneus/left IPL	40,7	−4.557	36	−57	48	285
Left IPL/PoCG	40,7,6,3	−4.234	−48	−51	36	200
**Altered AF in the slow-2 (0.167–0.25 Hz) band (LBLP** ***vs***. **HC)**						
Right cerebellum posterior lobe and brainstem		6.171	21	−45	−57	408
Left MTG/ITG	20,21	6.750	−63	−12	−27	153
Right aINS/fO	47	6.357	−9	12	−24	327
Right MTG/ITG	20,21	7.019	54	−12	−27	154
Left superior frontal gyrus	10	−5.013	−27	48	33	162
Right superior frontal gyrus	10	−4.806	9	63	24	105

Note: AF = Amplitude of fluctuations; BA = Brodmann area; LBLP = low back and leg pain; MNI = Montreal Neurological Institute.

### Interactions between disease status and the five specific frequency bands

**Figure 4:**
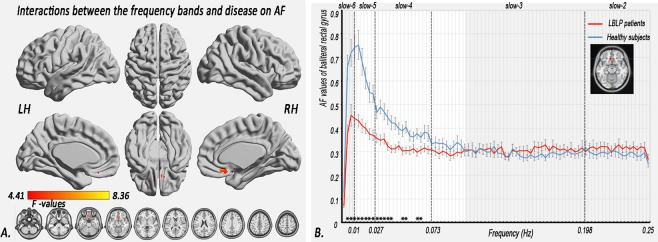
Interactions between the five specific frequency bands and disease status in relation to the AF. (**A**) Interaction between the specific frequency bands (slow-2 to slow-6) and group (LBLP patients and HCs) based on the ANOVA (flexible factorial design, 2 × 5, two-tailed, voxel-level P < 0.01, GRF correction, cluster-level P < 0.05). (**B**) Trend of AF in the rectal gyrus across the full-frequency band (0–0.25 Hz).

A significant interaction was identified between the frequency bands and disease status in the bilateral rectal gyrus (Fig. 4A, Table 2) *(two-tailed*, *voxel-level P* < *0*.*01*, *GRF correction*, *cluster-level P* < *0*.*05)*, where there were significant decreases in the AF in the slow-6, slow-5 and slow-4 bands, but slight increases in the AF in the slow-3 and slow-2 bands in the LBLP patients (Fig. 4B) *(two-tailed*, *voxel-level P* < *0*.*01*, *GRF correction*, *cluster-level P* < *0*.*05)*.

**Table 2:** Significant interactions between the AF of the five specific frequency bands and disease status (flexible factorial design, 2 × 5)

Brain regions	BA	Peak t-scores	MNI coordinates			Cluster size (voxels)
			x	y	z	
**Association between the frequency band (slow-2 to slow-6) and disease**						
Bilateral rectal gyrus	11	8.355^※^	6	30	−15	108

Note: ^※^The F-test was statistically significant for an interaction between the AFs of the five specific frequency bands (slow-2 to slow-6) and disease status. The T-test was statistically significant for particular analyses of interaction. All clusters were analyzed using a two-tailed test with a voxel-level threshold of P < 0.01, GRF correction and cluster-level of P < 0.05.

### Relationships between abnormal AF values and neuropsychological assessments

**Figure 5:**
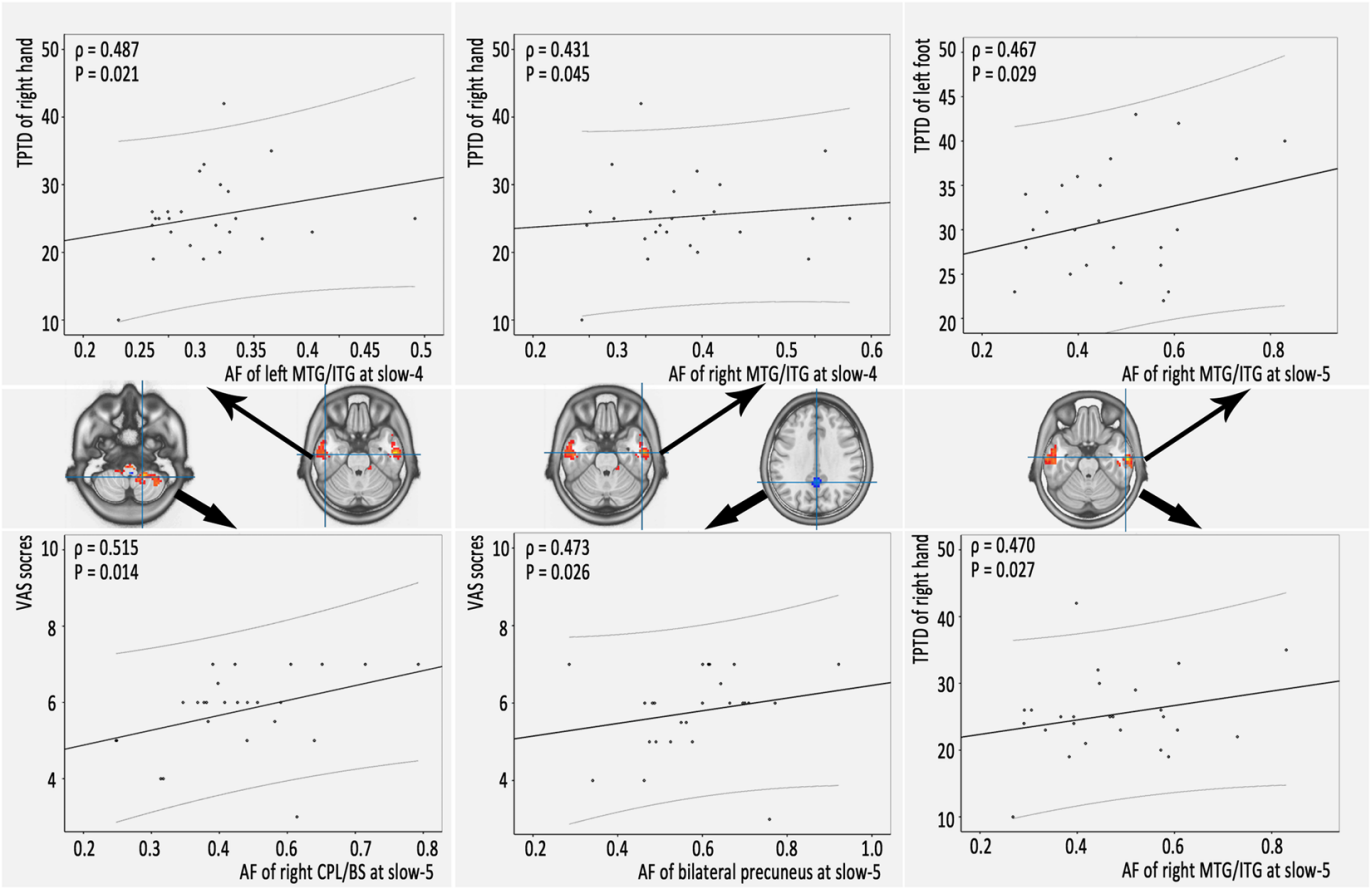
Clusters of altered AF of physiological significance in specific frequency bands (slow-4 and slow-5) were significantly correlated with the neuropsychological assessment scores in the LBLP patients (*P* < 0.05).

For correlational analyses, the AF values were extracted from brain areas showing significant differences between the two groups in the five specific frequency bands (slow-6, slow-5, slow-4, slow-3 and slow-2) and from the voxel-based AF comparisons. Partial correlations between the abnormal AF values and the neuropsychological assessment scores were calculated for the LBLP patients. Age, sex and mean FD were considered covariates of effects in this study. The results are shown in Fig. 5, Tables 3, 4 and S1–S3. In the LBLP patients, increases in AF in the slow-4 band in the left (ρ = 0.431, *P* = 0.045) and right MTG/ITG (ρ = 0.487, *P* = 0.021) were moderately positively correlated with the TPTD performance of the right hand. The AF in the slow-5 band in the right MTG/ITG was moderately positively correlated with TPTD performance in the left foot (ρ = 0.470, *P* = 0.027) and the right hand (ρ = 0.467, *P* = 0.029). An increase in the AF in the slow-5 band in the right CPL/BS (ρ = 0.515, *P* = 0.014) and a decrease in the AF in the bilateral PCUN (ρ = 0.473, *P* = 0.026) were moderately positively correlated with VAS scores. However, there were no significant associations after Bonferroni correction.

**Table 3:** Relationships between clinical indices and the AF of the slow-5 band in LBLP patients (ρ value/*P* value).

	Disease duration (months)	JOA scores	VAS scores	Sensory measurements				
				Fugl-Meyer scores	TPTD of right hand	TPTD of left hand	TPTD of right foot	TPTD of left foot
Right CPL/BS	0.374/0.086	−0.129/0.566	0.515/0.014*	0.040/0.860	0.162/0.472	−0.139/0.538	−0.306/0.166	−0.242/0.278
Left MTG/ITG	0.200/0.371	−0.047/0.834	0.237/0.289	0.100/0.657	0.341/0.120	0.194/0.387	0.035/0.876	0.081/0.719
Right MTG/ITG	0.090/0.691	−0.188/0.402	0.218/0.329	−0.081/0.720	0.470/0.027*	0.388/0.074	0.324/0.141	0.467/0.029*
Bilateral caudate/ACC	0.020/0.929	0.091/0.687	0.189/0.400	0.022/0.923	0.274/0.216	0.106/0.638	0.015/0.948	−0.036/0.875
Bilateral precuneus	0.136/0.547	−0.007/0.974	0.473/0.026*	−0.099/0.662	−0.159/0.481	−0.204/0.364	0.334/0.129	−0.223/0.320

Notes: CPL = cerebellum posterior lobe; JOA = Japanese Orthopaedic Association Back Pain Evaluation; TPTD = two-point tactile discrimination; VAS = visual analogue scale; *P < 0.05.

**Table 4:** Relationships between clinical indices and the AF of the slow-4 band in LBLP patients (ρ value/*P* value)

	Disease duration (months)	JOA scores	VAS scores	Sensory measurements				
				Fugl-Meyer scores	TPTD of right hand	TPTD of left hand	TPTD of right foot	TPTD of left foot
Right CPL/BS	0.212/0.343	−0.111/0.624	0.406/0.061	0.041/0.857	−0.017/0.940	−0.202/0.366	−0.288/0.193	−0.264/0.235
Right MTG/ITG	0.262/0.239	−0.029/0.898	0.292/0.187	0.034/0.880	0.431/0.045*	0.101/0.655	−0.018/0.938	0.089/0.695
Left MTG/ITG	0.385/0.077	0.047/0.836	0.286/0.228	0.131/0.562	0.487/0.021*	0.192/0.392	−0.109/0.628	−0.022/0.921
Bilateral BS/caudate/thalami	0.036/0.873	0.018/0.936	0.179/0.425	0.204/0.362	0.029/0.898	0.042/0.852	0.060/0.791	0.011/0.963
Right TOJ	−0.029/0.897	0.162/0.472	−0.005/0.983	−0.006/0.977	0.010/0.965	0.154/0.495	−0.004/0.985	−0.033/0.886
Right aINS/fO	0.002/0.992	0.229/0.306	0.124/0.582	0.041/0.855	0.215/0.337	0.220/0.325	−0.042/0.853	−0.033/0.884
Bilateral precuneus	−0.156/0.487	0.154/0.494	0.079/0.728	0.006/0.977	−0.123/0.586	−0.312/0.158	−0.354/0.106	−0.297/0.209
Left IPL/PoCG	0.105/0.643	0.154/0.493	0.310/0.160	0.018/0.938	−0.276/0.213	−0.197/0.380	−0.313/0.156	−0.261/0.241

Notes: aINS/fO = anterior insula/frontal operculum; JOA = Japanese Orthopaedic Association Back Pain Evaluation; TOJ = temporal–occipital junction; TPTD = two-point tactile discrimination; IPL/PoCG = inferior parietal lobule/postcentral gyrus; VAS = visual analogue scale; *P < 0.05.

## Discussion

The current study investigated the altered amplitudes of spontaneous BOLD fluctuations in different frequency bands in patients with LBLP. The LBLP patients showed significant differences in the AF in five specific frequency bands compared with HCs, which involved the pain matrix and sensory-processing brain regions. Importantly, we demonstrated that LBLP patients exhibited significantly increased oscillatory patterns in the full-frequency band (0–0.25 Hz) in the right CPL and the left and right MTG/ITG and significantly decreased oscillatory patterns in the right IPL/PoCG, bilateral CAU and bilateral PCUN. Moreover, there were pain-related alterations in the AF in the slow-5 band in the right CPL/BS and bilateral PCUN, which were related to the VAS pain scores. There were also sensory-related alterations in the AF in the slow-4 band in the left and right MTG/ITG and in the slow-5 band in the right MTG/ITG, which were related to performance on the TPTD test. These findings provide the full picture of the AF observed in BOLD waves, which is potentially useful for selecting specific frequencies to improve the detection of LBLP-related brain activity.

### Alterations of the AF in LBLP patients in specific frequency bands and full-frequency bands

In this study, we investigated frequency band-specific changes in spontaneous BOLD fluctuations in the resting state. We further demonstrated robust changes in the AF in the full-frequency bands (0–0.25 Hz) in the right CPL, bilateral PCUN, bilateral caudate CAU and right IPL/PoCG of LBLP patients. In previous studies, specific AF changes in intrinsic fluctuations or hemodynamic brain activity at different frequency bands have been observed in different physiological states, such as when the eyes are open or closed^21^, and in various disease states (e.g., amnestic mild cognitive impairment^26^, chronic primary insomnia^19^ and schizophrenia)^27^. In the above studies, the strategy of frequency division was advantageous, enabling the detection of significant differences in the AF in specific frequency bands, including high-frequency bands. Although the dynamic oscillations of high-frequency bands are not fully understood, these oscillations may originate from the neuronal discharge of pyramidal cells as the receptive field “enslaves” the basket cells through resonance tuning^7^. There is also growing evidence that high-frequency fluctuations of BOLD waves can be modulated by different stimuli and are not simply noise but may be of physiological importance. Therefore, there is reasonable evidence that the altered regions of AF in both relatively low- and high-frequency bands may be of physiological importance and may reflect modulations as a result of the disease state in LBLP patients.

The aINS/fO, CAU, PoCG and CPL are involved in pain processing. Human brain imaging studies have revealed multiple pathways and several discrete brain regions that are involved in pain processing. Afferent nociceptive information enters the brain from the spinal cord and travels through the spinocerebellar, spinothalamic-insula, spinothalamic-S1 and spinoparabrachio-amygdaloid-basal ganglia pathways^28^. In the pain matrix, there are decreases in the cortical density^6,7^ and thickness^7^ and a change in the white matter integrity^2–4^ in the aINS/fO. Changes in insular volume may contribute to the transition from acute to chronic pain^5^. S1 (PoCG) is a component of the lateral pain system that encodes sensory-discriminative aspects of pain perception. Patients with low-intensity LBLP show decreased functional connectivity of S1 compared with matched HCs, and when chronic LBLP patients experience high-, rather than low-intensity pain, the functional connectivity at S1 increases. These functional changes in S1 may exist in the context of structural plasticity (increased cortical thickness) in chronic LBLP patients^8^. Paresthesia or numbness, a “tingling, pricking” sensation, with other sensory modalities activating SI/SII regions, may result in cognition by engaging mutual cortical-level processing^29,30^. In the current study, the increased AF in the right CPL and right aINS/fO and the decreased AF in the bilateral CAU and PoCG imply that there are differences in pain perception depending on the context and meaning of pain. The decreased AF observed in the LBLP patients may be due to diminished sensory input as a result of paresthesia (numbness) of the lower extremities.

In this study, we also observed an increase in the AF in the bilateral MTG/ITG in the LBLP patients. The MTG and ITG are involved in the visual processing of distance-related information and spatial awareness, respectively. In patients with low back pain, the MTG and ITG may be activated by somatosensory stimuli^31,32^. In the current study, the increased AF in the left or right MTG/ITG was correlated with the TPTD performance of the right and left feet, indicating functional compensation or plasticity in parts of the spatial sensory-processing regions.

We also found that the altered regions of the bilateral PCUN and left IPL consistently showed low- and high-frequency bands. The PCUN and IPL belong to the DMN, and disrupted connectivity of the DMN has been reported in previous studies of low back pain, implying dynamic impairment beyond the feeling of pain^11–14^. Altered neuronal activity of the DMN has been demonstrated in chronic low back pain patients during external stimulation and in the resting state and can be functionally reversed by acupuncture treatment^33^. More recent rs-fMRI studies have shown that connectivity within the DMN is altered in other chronic pain conditions, including migraines^34^, fibromyalgia^25^, chronic somatic pain^23^ and diabetic neuropathic pain^35^. Therefore, the DMN has been proposed as a biomarker for several chronic pain conditions^36^.

### Interaction between frequency and disease changes in LBLP patients

In this study, significant interactions were identified between the five specific frequency bands and disease status in the bilateral rectal gyrus, suggesting that different frequency bands may have specific pathological relevance in this region. In the bilateral rectal gyrus, there were significant decreases in the AF in the slow-6, slow-5 and slow-4 bands and slight increases in the AF in the slow-3 and slow-2 bands. In a previous study, Baliki *et al*.^12^ observed a graded shift phenomenon of power from low- to high-frequency bands in the visual ventral stream, suggesting that a closed relationship exists between the anatomical structures and the full spectrum of intrinsic oscillations. Lower-frequency fluctuations reflect long-distance neural activity due to a higher magnitude of power, while higher-frequency fluctuations reflect local neural activity due to a lower magnitude of power^20,37,38^. Evidence from neuroimaging and neuropsychology studies shows that the rectal gyrus is important in processing rewards and punishments and in linking pleasant and painful touch sensations^39^. Therefore, the findings of the current study may suggest that LBLP patients exhibit a reduced ability to process long-distance neural activity and an increased ability to process local neural activity in the rectal gyrus, hinting that painful inhibition by reward appears to distinguish neural representations. However, this assumption needs to be confirmed in future studies.

### Correlations between clinical indices and an abnormal AF in specific frequency bands

The correlational analyses showed that the changes in AF in the slow-5 band were related to the VAS pain scores, while the sensory-related changes in AF in the slow-4 and slow-5 bands were related to TPTD performance. The cerebellum plays a cross-modal modulatory role in the nociceptive processing of pain through electrical and/or chemical means and the processing of pain corresponding to emotion, cognition and motor control^40,41^. Recent studies have demonstrated increased activity and functional connectivity between the cerebellum, brainstem and periaqueductal grey matter during stimulation of nociception, involving the descending antinociceptive network^41^.

In this study, a positive correlation was found between a decreased AF of the bilateral PCUN and pain intensity (VAS scores) in LBLP patients. This finding indicates that when LBLP patients feel higher pain, they have relatively little ability to reduce the AF value in the PCUN. Does this finding seem to be contradicted by previous studies? Many studies suggest that pain-induced DMN deactivations or hypo-activity is negatively correlated with pain intensity^34,35,42^. However, as a key area of the DMN, the PCUN did not contribute to the actual representation of pain and mainly predicted individual differences in sensitivity to pain. In the resting state, the patients spend nearly half of their time on mind wandering to cope with pain, while they are subjected to increased dynamic functional communication with the region mediating pain suppression^43^. One possible explanation is that the PCUN dysfunction observed in this study might underlie or be related to a reduced ability to pay attention. In short, an increased AF of the right CPL/BS and decreased AF of the PCUN in these pain-related regions may reflect maladaptive pain processing that influences affective responses to pain experiences^34^. A previous study of nociceptive and stolidity stimuli in LBLP patients revealed similar responses in the ipsilateral and contralateral S2 regions, which integrate inputs across multiple sensory modalities^44,45^. In this study, the TPTD test was used to assess the tactile spatial resolution, and the performance of the LBLP patients indicated that these patients exhibited hyperesthesia. The MTG/ITG is associated with visual stimuli processing for distance-related information and spatial awareness. In this study, although the MTG/ITG, and not S2, was associated with TPTD performance, we believe that these results may provide evidence of clinical measurements related to functional plasticity in areas that exhibit dynamic oscillatory waves of BOLD signals in LBLP patients.

This study has several technical and biological limitations that must be acknowledged. First, this study involved mostly middle-aged to elderly LBLP patients. Due to factors such as brain atrophy in the elderly population, only patients younger than 65 years of age were included in the AF analyses. Second, for statistical consistency, P < 0.01 was set for the voxel- and ROI-level analyses; however, the AF values extracted for ROIs were mean values from overlapping regions of the five specific frequencies. Therefore, caution should be exercised in interpreting the comparison of AF between groups in the five specific frequency bands and the 84 narrow band bins. Third, all of the LBLP patients presented pain and numbness, or “dual-stimulation”, as characteristics of this cohort. However, other features of this “dual-stimulation” (i.e., pain, but not numbness, and vice versa) have not been characterized. Therefore, in this study, the observed moderate relationships may be generically related to the chronic pain and/or paresthesia state without multiple comparisons, and it is likely that one or more false discoveries or type I errors are made; however, the findings are interesting nevertheless. When interpreting the results, the fact that the brain regions where AF is reduced are not the same regions that are associated with TPTD in the correlational analyses should be addressed at some level. Finally, this study is limited by its small sample size and the fact that 23 (92%) of the patients also presented with both pain and numbness, which should be considered when interpreting the results in the analyses of TPTD and specific AF correlations. In the near future, we will divide our patient sample by a median split for VAS scores (high/low) to determine whether the broad patterns of increases and decreases in the AF compared with the HCs hold for both groups.

## Conclusions

In this study, we showed that LBLP patients exhibit alterations of the AF in both low and high ranges of frequency bands involving the pain matrix and sensory-processing regions. Moreover, in all specific frequency bands, the pain-related alterations in the AF were frequency-dependent in several brain regions, which was related to clinical indices. These findings suggest that specific frequency ranges, even high-frequency bands, should be selected for the detection of pain-related intrinsic activity in future studies of LBLP patients.

## Methods

This case-control study was approved by the Medical Research Ethics Committee and the Institutional Review Board of the First Affiliated Hospital of Nanchang University. All subjects provided written informed consent and were identified only by number before undergoing an MR scan. All of the research procedures were performed according to the ethical principles of the Declaration of Helsinki and approved guidelines.

### Subjects

From Oct. 2016 to Jul. 2017, a cohort of 61 participants, including LBLP patients and age-, sex- and education-matched HCs, were recruited from our hospital and from the local community. All patients were clinically diagnosed using available computed tomography (CT) and/or MRI images. Patients were included if they had more than 1 ruptured annulus fibrosus with compressed soft tissue, resulting in low back and leg pain for at least the last month that had failed to respond to conservative treatment with medications, exercise and physical therapy^15^. The inclusion criteria were as follows: (1) clear evidence of discogenic compression in a lumbar CT and/or MRI; (2) radiating pain (VAS score > 4) from the lumbar region to the buttock(s) and lower limb(s) that increases with abdominal pressure (e.g., coughing, sneezing); (3) a positive straight-leg raise test and pick-up test and weak or non-existent patellar and Achilles tendon reflexes; (4) self-reported failure of improvement of pain with non-steroidal anti-inflammatory drugs (NSAIDs, e.g., Motrin, Advil and Naproxen) and acetaminophen (e.g., Tylenol) without opioids; and (5) an age of 35–65 years and volunteering to enroll in the study. The exclusion criteria were as follows: (1) spinal canal compression due to trauma or infection; (2) previous spinal surgery or pre-existing spinal abnormality; (3) other neurological disorders, such as a history of stroke, epilepsy, Parkinson’s disease, dementia or multiple sclerosis; (4) cardiovascular, cerebrovascular, liver, kidney or hematopoietic diseases; (5) the presence of calcifications on a spinal protrusion, lateral recess stenosis, spinal stenosis, pyriformis syndrome or sciatica; and (6) diagnosis of LDH without clinical symptoms. In addition, participants who refused or had contraindications for MRI, such as claustrophobia or metallic implants or devices, were excluded. Participants whose resting-state fMRI (rs-fMRI) revealed head motion with maximum displacement in the orthogonal direction (x, y, z) > 3 mm or maximum rotation (x, y, z) > 3.0° were excluded.

### Clinical evaluation

All subjects underwent the following clinical evaluations before the MRI: the VAS (0–10) for pain, the JOA Back Pain Evaluation questionnaire (−6 to 29) to examine the impact of neuropathic or nociceptive pain on quality of life^46^, the Barthel index (0–100) for performance in activities of daily living and the TPTD test to assess the tactile spatial resolution ability^47^.

### MRI data acquisition

All subjects underwent MRI in a Trio 3.0-tesla Siemens whole-body scanner with an 8-channel head coil (Trio, Siemens, Munich, Germany). For rs-fMRI, the following settings were used: repetition time (TR)/echo time (TE) = 2000/30 ms, matrix = 64 × 64, field of view (FOV) = 220 × 220 mm, 30 interleaved axial slices, 4 mm thickness, inter-slice gap of 1.2 mm and 240 volumes in 8 min. During rs-fMRI, the subjects were asked to keep their eyes closed, clear their mind and not fall asleep. A 3-dimensional, high-resolution T_1_-weighted magnetization-prepared rapid gradient-echo (MP-RAGE) sequence was employed to obtain anatomical images using the following settings: TR/TE = 1900 ms/2.26 ms, FOV = 215 mm × 230 mm, matrix = 240 × 256 and 176 sagittal slices at a 1.0 mm slice thickness with no gaps.

Additional conventional T_2_-weighted and T_2_-fluid-attenuated inversion recovery (FLAIR) sequences were acquired for the diagnosis of LBLP. Sagittal and axial conventional T_1_-weighted, T_2_-weighted and T_2_-fat suppression sequences were acquired for the lumbar spine and discs from L1 to S3. At the end of the scanning sessions, all participants reported that they had not fallen asleep during the scan according to the Epworth Sleepiness Scale (ESS) questionnaire.

### Image preprocessing

The preprocessing of rs-fMRI images was performed using a toolbox for Data Processing & Analysis of Brain Imaging^48^ (http://rfmri.org/dpabi) based on statistical parametric mapping (SPM12, http://www.fil.ion.ucl.ac.uk/spm/software/spm12/), which was run on MATLAB 8.4.0 (Mathworks, Natick, MA, USA). The main preprocessing steps included the following: (1) after format transformation (dicom to nifti), the first 10 images were discarded for stabilization; (2) the remaining 230 images underwent slice timing, spatial realignment and voxel-specific head motion calculations (head motion was < 3 mm of translation and < 3.0° of angular rotation along any axis); (3) the group differences in head motion were evaluated according to the criteria described by Jenkinson *et al*. (including criterion: framewise displacement < 0.2 mm)^49^; (4) the high-resolution individual T_1_-weighted images were co-registered to the mean functional image using a linear transformation; (5) the Montreal Neurological Institute (MNI) space was transformed with the Diffeomorphic Anatomical Registration Through Exponentiated Lie algebra (DARTEL) tool and was re-sampled as 3 × 3 × 3 mm^3^ cube voxels; (6) spatial smoothing was performed with a 6-mm full-width-half-maximum Gaussian kernel; and (7) to reduce the effects of head motion and non-neuronal BOLD fluctuation, after linear detrending, nuisance linear regression was performed for the white matter, the cerebrospinal fluid, the global signal, six head motion parameters at a single time point and at one time point earlier and the 12 corresponding squared items (Friston 24-parameter model).

### Temporal filtering and AF analysis

(1) Predefined settings were used to calculate the AF in the five frequency bands. According to the Buzsáki framework^7^, the preprocessing fMRI data were divided into five specific frequency bands: slow-6 (0–0.01 Hz), slow-5 (0.01–0.027 Hz), slow-4 (0.027–0.073 Hz), slow-3 (0.073–0.198 Hz) and slow-2 (0.198–0.25 Hz).

(2) Predefined settings were used to characterize the changes in the AF with the evolution of frequencies in the full-frequency bands. The preprocessing fMRI data were divided into 84 narrow band bins with each narrow frequency band covering 0.003 Hz^50^.

(3) The AF was defined as the sum of the amplitudes within a predefined frequency domain using the fast Fourier transform procedure (FFT; taper percent = 0, FFT length = shortest) for each voxel’s time series, extended from the calculation of the amplitude of low-frequency fluctuations^51^. The AF was transformed into Fisher-z values for voxel-wise group analyses. The calculation of the AF was performed using the Data Processing & Analysis of Brain Imaging toolbox.

### Statistical analyses

To explore the differences in the AF between the LBLP and HC groups in the five specific frequency bands, a second-level random-effect two-sample *t*-test was performed on individual z-AF maps in a voxel-by-voxel manner. To calculate the correlation between the specific frequency band and group in the AF, we performed an ANOVA (flexible factorial design, 2 × 5) using SPM12 with the groups (LBLP and HC) as a between-subject factor and the frequency band (slow-2 to slow-6) as an independent measure. All significant clusters were reported on the MNI coordinates, and T-values of the peak voxel were determined. Significance was determined at a voxel-level threshold (*P* < 0.01) with the GRF theory correction at a cluster-level threshold (two-tailed, *P* < 0.05).

The main brain regions showing an altered AF in the LBLP versus HC groups were identified as the ROIs for subsequent full-frequency band analyses. The AF values of each ROI were extracted from 84 amplitude maps with narrow frequency bands. Two-sample *t*-tests were performed in each narrow band bin (Bonferroni-corrected α level of *P* < 0.00059, 0.05/84 dependent variables).

Comparisons of clinical characteristics and indices between the two groups were conducted using two-sample *t*-tests and *χ*^2^-tests in SPSS (release 13.0, SPSS Inc., Chicago, IL, USA). In addition, partial correlational analyses were performed between the clinical indices (JOA, VAS, etc.) and the AF values for brain areas that exhibited significant differences between the LBLP and HC groups using the SPSS 13.0 software program (*P* < 0.05); the effects of age, sex and mean FD were considered. Bonferroni corrections were employed to correct for type I error across analyses (0.05/n dependent variable; two-tailed α level of *P* < 0.05/n. Note: n is the number of brain areas showing significant differences in LBLP patients).

## Supplementary information

Supplementary table

## Data Availability

The datasets generated during the current study are available from the corresponding author upon reasonable request.
