# Supplementary material for: Compressing the lumbar nerve root changes the frequency-associated cerebral amplitude of fluctuations in patients with low back/leg pain
Source: Sci Rep. 2019 Feb 19;9:2246. doi: 10.1038/s41598-019-38721-5 (PMC6381144; doi:10.1038/s41598-019-38721-5)
Supplement: Supplementary file 1 — Supplementary table [file 41598_2019_38721_MOESM1_ESM.docx]

**Compressing the lumbar nerve root changes the frequency-associated cerebral amplitude of fluctuations in patients with low back/leg pain**

Fuqing Zhou^1,5^, Yanlin Zhao^1,5^, Li Zhu^2^, Jian Jiang^1,5^, Muhua Huang^1,5^, Yong Zhang^2^, Ying Zhuang^4^, and Honghan Gong^1,5^

^1^Department of Radiology, The First Affiliated Hospital, Nanchang University, Nanchang, 330006, People’s Republic of China; ^2^School of Information Engineering, Nanchang University, Nanchang, 330031, People’s Republic of China; People’s Republic of China; ^3^Department of Pain Clinic, The First Affiliated Hospital, Nanchang University, Nanchang, Jiangxi Province, 330006, ^4^Department of Oncology, The Second Hospital of Nanchang, Nanchang, 330003, People’s Republic of China; ^5^Neuroimaging Lab, Jiangxi Province Medical Imaging Research Institute, Nanchang, 330006, People’s Republic of China.

**Supplementary Materials**

***Table S1.*** *The relationship between clinical indices and the AF in the slow-6 band in the LBLP patients (ρ values/P values)*

|  | Disease duration (months) | | JOA scores | | VAS scores | | Sensory measurements | | | | | | | | | |  |
| --- | --- | --- | --- | --- | --- | --- | --- | --- | --- | --- | --- | --- | --- | --- | --- | --- | --- |
|  |  |  |  |  |  |  | Fugl-Meyer scores | | TPTD of right hand | | TPTD of left hand | | TPTD of right foot | | TPTD of left foot | | |
| Right MTG/ITG | 0.076/0.731 | -0.055/0.802 | | -0.006/0.978 | | 0.129/0.557 | | 0.608/0.002* | | 0.664/0.001* | | 0.536/0.008* | | 0.557/0.006* | |  |  |
| Left MTG/ITG | 0.111/0.615 | -0.079/0.722 | | 0.194/0.375 | | -0.057/0.797 | | 0.484/0.019* | | 0.329/0.125 | | 0.201/0.359 | | 0.232/0.287 | |  |  |
| Bilateral BS/caudate/thalami/ACC | -0.094/0.671 | -0.209/0.339 | | 0.142/0.518 | | -0.076/0.731 | | 0.109/0.622 | | 0.147/0.503 | | 0.158/0.473 | | 0.393/0.063 | |  |  |
| Bilateral precuneus | 0.015/0.946 | 0.088/0.688 | | 0.004/0.987 | | -0.186/0.395 | | 0.106/0.630 | | 0.162/0.461 | | -0.045/0.840 | | -0.170/0.439 | |  |  |

*Notes: ACC=anterior cingulate cortex; BS=brainstem; JOA=Japanese Orthopaedic Association; MTG/ITG=middle and inferior temporal gyrus; TPTD=two-point tactile discrimination; VAS=visual analogue scale; * P<0.05.*

***Table S2.*** *The relationship between clinical indices and AF of the slow-3 band in LBLP patients (ρ values/P value)*

|  | Disease duration (month) | JOA scores | VAS scores | Sensory measurement | | | | | | |  |
| --- | --- | --- | --- | --- | --- | --- | --- | --- | --- | --- | --- |
|  |  |  |  | Fugl-Meyer scores | TPTD of right hand | | TPTD of left hand | TPTD of right foot | TPTD of left foot | | |
| Right CPL/BS | 0.334/0.129 | -0.184/0.415 | 0.668/0.001* | -0.150/0.504 | 0.060/0.792 | -0.222/0.320 | | -0.353/0.107 | | -0.113/0.616 |  |
| Left MTG/ITG | 0.215/0.337 | 0.019/0.932 | 0.143/0.525 | -0.061/0.789 | 0.603/0.003* | 0.276/0.214 | | 0.142/0.530 | | 0.243/0.276 |  |
| Right MTG/ITG | 0.159/0.481 | -0.219/0.369 | 0.129/0.568 | -0.102/0.651 | 0.507/0.016* | 0.186/0.408 | | 0.222/0.320 | | 0.301/0.173 |  |
| Bilateral rectal gyrus | 0.270/0.213 | -0.095/0.665 | 0.343/0.109 | -0.370/0.083 | -0.152/0.490 | -0.077/0.728 | | -0.063/0.776 | | 0.015/0.947 |  |
| Bilateral caudate/thalami | 0.160/0.478 | -0.061/0.787 | 0.465/0.029* | -0.074/0.744 | 0.200/0.373 | 0.027/0.905 | | 0.060/0.792 | | 0.186/0.408 |  |
| Right aINS/fO | 0.309/0.162 | -0.011/0.961 | 0.394/0.070 | -0.088/0.698 | 0.264/0.236 | 0.226/0.312 | | -0.011/0.960 | | 0.175/0.437 |  |
| Right IPL | 0.407/0.060 | 0.199/0.375 | 0.376/0.084 | 0.115/0.609 | -0.086/0.703 | -0.021/0.926 | | -0.135/0.549 | | -0.100/0.658 |  |
| Bilateral precuneus | 0.112/0.620 | 0.393/0.070 | 0.050/0.825 | 0.180/0.424 | 0.110/0.626 | 0.387/0.075 | | 0.210/0.347 | | 0.251/0.260 |  |
| Left IPL/PoCG | 0.249/0.263 | 0.267/0.229 | 0.207/0.355 | 0.229/0.305 | -0.068/0.763 | 0.126/0.578 | | 0.035/0.876 | | 0.084/0.710 |  |

*Notes: JOA=Japanese Orthopaedic Association; TPTD=two-point tactile discrimination; VAS=visual analogue scale; * P< 0.05.*

***Table S3.*** *The relationship between clinical indices and AF of the slow-2 band in LBLP patients (ρ values/P value)*

|  | Disease duration (month) | JOA scores | VAS scores | Sensory measurement | | | | | | |  |
| --- | --- | --- | --- | --- | --- | --- | --- | --- | --- | --- | --- |
|  |  |  |  | Fugl-Meyer scores | TPTD of right hand | | TPTD of left hand | TPTD of right foot | TPTD of left foot | | |
| Right CPL/BS | 0.298/0.192 | -0.425/0.048* | 0.618/0.002* | -0.226/0.313 | 0.088/0.698 | -0.096/0.672 | | -0.177/0.431 | | 0.059/0.795 |  |
| Left MTG/ITG | 0.125/0.580 | 0.051/0.821 | 0.119/0.597 | -0.064/0.778 | 0.521/0.013* | 0.274/0.218 | | 0.204/0.363 | | 0.242/0.277 |  |
| Right aINS/fO | 0.1025/0.652 | -0.202/0.367 | 0.303/0.170 | -0.384/0.078 | 0.244/0.274 | 0.350/0.110 | | 0.261/0.242 | | 0.419/0.052 |  |
| Right MTG/ITG | 0.168/0.455 | -0.049/0.830 | 0.030/0.896 | -0.043/0.849 | 0.496/0.019* | 0.191/0.393 | | 0.309/0.161 | | 0.294/0.184 |  |
| Left SFG | 0.510/0.015* | 0.178/0.427 | 0.189/0.400 | 0.128/0.569 | 0.210/0.349 | 0.100/0.659 | | -0.071/0.753 | | -0.153/0.490 |  |
| Right SFG | 0.497/0.018* | 0.174/0.437 | 0.185/0.407 | 0.218/0.368 | 0.214/0.352 | 0.108/0.654 | | -0.074/0.758 | | -0.151/0.493 |  |

*Notes**: JOA=Japanese Orthopaedic Association; SFG=superior frontal gyrus; TPTD=two-point tactile discrimination; VAS=visual analogue scale; * P< 0.05.*
